# 3D monitors improve performance on the HUGO™ RAS system: a randomised trial

**DOI:** 10.1007/s00464-024-11275-y

**Published:** 2024-10-03

**Authors:** Theresa Bruun Østdal, Diana Hai Yen Tang, Rikke Groth Olsen, Louise Møller Olsen, Lars Konge, Flemming Bjerrum

**Affiliations:** 1 Copenhagen Academy for Medical Education and Simulation, Centre for HR and Education, The Capital Region, Ryesgade 53B, 2100 Copenhagen, Denmark; 2 The Faculty of Health Sciences, University of Southern Denmark, Odense, Denmark; 3 Department of Clinical Medicine, Faculty of Health and Medical Sciences, University of Copenhagen, Copenhagen, Denmark; 4 Urological Research Unit, Department of Urology, Centre for Cancer and Organ Diseases, Copenhagen University Hospital - Rigshospitalet, Copenhagen, Denmark; 5 Gastrounit - Surgical Section, Copenhagen University Hospital - Amager and Hvidovre, Hvidovre, Denmark

**Keywords:** Robotic surgery, Simulation, Training, HUGO, Three-dimensional, Two-dimensional

## Abstract

**Background:**

Robot-assisted surgery is used worldwide, allowing surgeons to perform complex surgeries with increased precision and flexibility. It offers technical benefits compared to traditional laparoscopic surgery due to its utilization of both 3D vision and articulated instruments. The objective was to investigate the isolated effect of 3D- versus 2D monitors when working with articulated instruments in robot-assisted surgery.

**Methods:**

Surgical novices (medical students, *n* = 31) were randomized to simulation-based training with either the 3D vision switched on or off. Both groups completed each of the four exercises six times over two sessions on the Medtronic Hugo™ RAS system simulator. The outcome was the simulator performance parameters and a visual discomfort questionnaire.

**Results:**

For the efficiency parameters, we found that both groups improved over time (*p* < 0.001) and that the intervention group (3D) consistently outperformed the control (2D) group (*p* < 0.001). On the other hand, we didn’t find any significant difference in the error metrics, such as drops (*p*-values between 0.07 and 0.57) and instrument collisions (*p*-values between 0.09 and 0.26). Regarding Visual Discomfort, it was significantly more difficult for the 3D group to focus (*p* = 0.001).

**Conclusion:**

3D monitors for an open robotic console improve efficiency and speed compared to 2D monitors in a simulated setting when working with articulated instruments.

**Supplementary Information:**

The online version contains supplementary material available at 10.1007/s00464-024-11275-y.

The use of robot-assisted surgery has increased exponentially over the last decade [1]. Robot-assisted surgery has several technical advantages over conventional laparoscopy, especially due to the combination of 3D vision and articulated instruments. These advantages provide better depth perception/spatial location, more degrees of freedom of instruments, and more versatile instrument movements.

Several studies have compared the effect of 3D versus 2D by comparing robot-assisted surgery with laparoscopy and found it improves performance [2, 3]. However, these studies did not differentiate between the effect of articulated instruments and 3D vision.

Only a few studies have examined the isolated effect of 3D vision when using robotic surgical systems. They found it to reduce operating time and improve surgical performance, but they have only assessed the da Vinci® Surgical system (Intuitive Surgical, Sunnyvale, CA) [4–6].

Newer 3D monitors with built-in ultra-high-definition (4 K) technology indicate that these 3D vision systems reduce task performance time by improving in-depth perception, spatial location, precision, and instrument handling [7, 8]. There has also been a development in the design of robotic surgical systems with the introduction of multiple new robotic systems. One of the newer systems, the Hugo™ RAS system, has an open console design with a screen and 3D glasses, which differs from the da Vinci® Surgical system, which has a closed console [9]. With the open console design, you can turn on or off the 3D effect, depending on the surgeon's preference, as some surgeons may experience side effects such as nausea, oculomotor, and disorientation when using 3D monitors [10]. No studies have examined the effect of 3D monitors on open console systems. This trial aimed to investigate the effect of 3D monitors when working in an open console system with articulated instruments in robot-assisted surgery.

## Method

The trial was a single-centre, randomised trial following the CONSORT statement [11]. It was submitted to The Regional Ethics Committee, which found no ethical approval necessary (ID: 22025389). All participants received verbal and written information before giving written informed consent to participate.

### Setting

Data collection was done at the Copenhagen Academy for Medical Education and Simulation (CAMES), Copenhagen, Denmark.

### Participants

Participants were medical students from the University of Copenhagen. They were recruited by an online post in a Facebook group for medical students. Inclusion criteria were: (1) Being enrolled as a medical student at the University of Copenhagen; (2) Giving signed informed consent. Exclusion criteria were: (1) Having participated in prior studies or similar involving robot-assisted training; (2) Having hands-on experience with robot-assisted surgery; (3) Did not speak Danish on a conversational level.

### Randomization and blinding

Before randomization, all participants received a unique trial identification number. Participants were randomized using the web-based system Sealed Envelope (London, United Kingdom) [12]. We used a 1:1 randomization ratio using a random permuted blocks sequence concealed from the investigators. Participants were tested for stereo acuity using the TNO test (plates V and VI). The TNO test was administered by having participants wear red-green spectacles and look at the two plates containing optotypes shaped as discs with a sector missing. The optotypes covered four different depth levels ranging from 60 to 480 arcsec. Participants were stratified as either having stereo blindness (a score of 480 or above) or not having stereo blindness (a score below 480) [13]. All participants were blinded to their allocation. It was impossible to blind investigators, but the data were automatically captured and stored on the simulator.

### Simulator and exercises

**Fig. 1:**
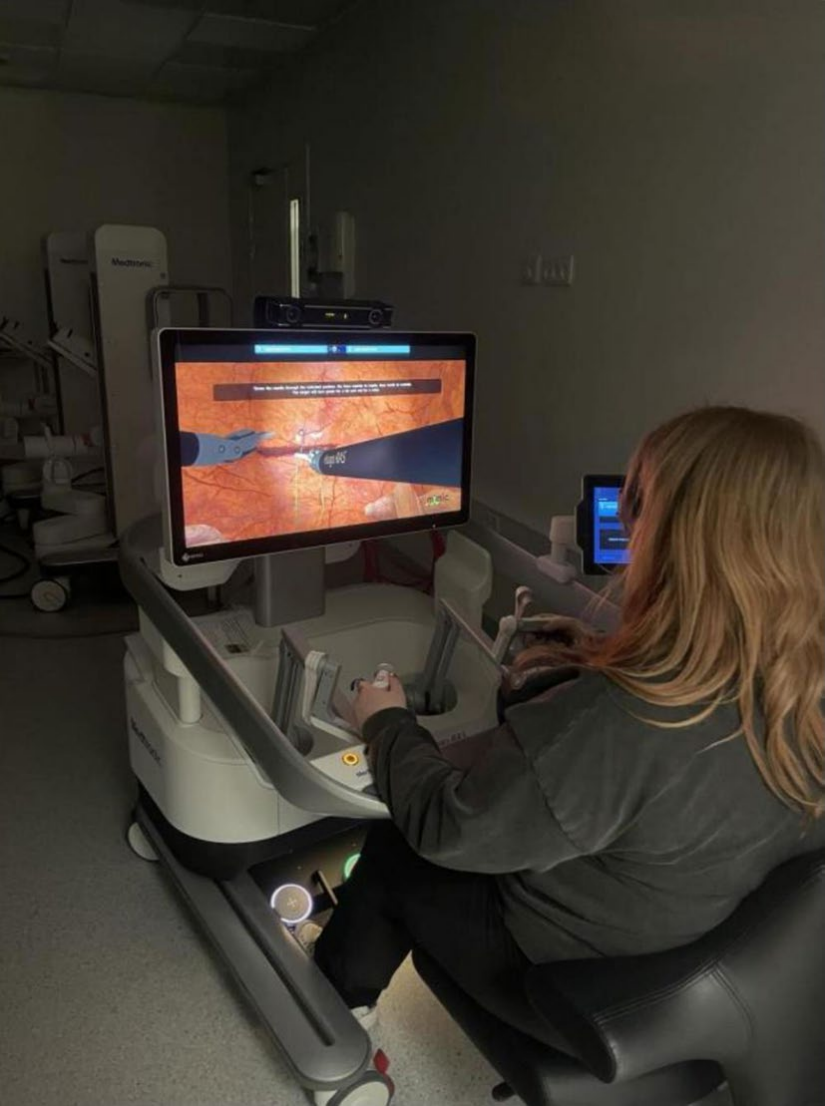
Photo of the Hugo simulator setup

We used the Medtronic Hugo RAS™ simulator connected to a Hugo console (Fig. 1). The console consists of one screen with a set of 3D glasses. All participants in the study had to wear 3D glasses as the glasses have sensors on the frame, which are detected by the console to ensure the surgeon does not perform surgery while looking away. Without these glasses, performing any tasks on the robotic system is impossible. The simulator has two Easy-grip controllers to control instruments and navigate the camera and seven-foot pedals through which the participants can control the robotic arms and activate energy devices. It was possible to adjust the height of the simulator to ensure the most ergonomically correct working position. The four exercises used were Peg Board II, Thread the Rings II, Ring Tower Transfer, and Suturing Wound—horizontal.

### Intervention

All participants were equipped with 3D glasses before entering the simulator room and could not take them off until they left the room. The participants were randomized to train under either 3D- (intervention group) or 2D conditions (control group). One of the investigators turned the 3D effect on or off before the participants entered the room. This ensured the participants couldn't decipher the randomization by removing the glasses inside the room. After that, one of the investigators introduced them to the proper use of the simulator and instrument handling. They were allowed one attempt on each of the four exercises to familiarize themselves with the simulator and the exercises. Participants performed two rounds of the four exercises during the first session. Participants came back for a second session, where they performed three more rounds of the exercises. All exercises were performed in the same order. During the second session, participants followed the same protocol for the 3D glasses. Participants could participate in a maximum of one session per day. After both sessions, the participants answered a visual and physical discomfort questionnaire. After the second session, the participants were also asked whether they believed they had practiced with the 3D effect turned on or off. The questionnaire was answered by the participants outside of the simulator room.

After enrolment, participants were not allowed to participate in robot-assisted surgery or similar studies until they had completed their participation. An investigator was present during training to assist with technical problems but was not otherwise allowed to help participants or give feedback.

### Outcome measures

We compared the two groups' performance based on simulator-generated metrics for each exercise. The secondary outcome was the visual and physical discomfort questionnaire, measured after each training session. The questionnaire lists the possible adverse symptoms reported in the literature when using 3D vision: eye strain, headache, dizziness, nausea, tiredness, and difficulty focusing. Participants listed their symptoms on a scale from 1 to 5, with one being the lowest.

The participants could also add other symptoms not mentioned in the questionnaire.

### Sample size calculation

We used a convenience sample due to the lack of existing data, and because we used a repeated measurement design, calculating the sample size was impossible. Based on our experience with other simulation-based interventions, we opted to include a minimum of 15 participants in each group.

### Statistical analysis

Intergroup comparisons over time for continuous variables were analyzed using mixed two-way-repeated measurement ANOVA after LOG-transformation of the data if they fulfilled the required assumptions. Continuous variables that did not meet the assumptions were analyzed using a dependent samples* t*-test for each group, separately comparing the first and fifth attempts.

Non-continuous variables (e.g., error variables like instrument collisions, missed targets, drops, and tower knockoffs) were analyzed by calculating the cumulative number of errors over all five attempts for each participant and the groups compared using the Mann–Whitney *U* test. The secondary outcome (Visual and physical discomfort questionnaire) was also analyzed using the Mann–Whitney *U* test. A significance level of *p* < 0.05 was used. Fisher’s exact test was used to examine the effect of the blinding procedure. Analysis was done using SPSS® version 28.0 (IBM, Armonk, NY, USA).

## Results

**Fig. 2:**
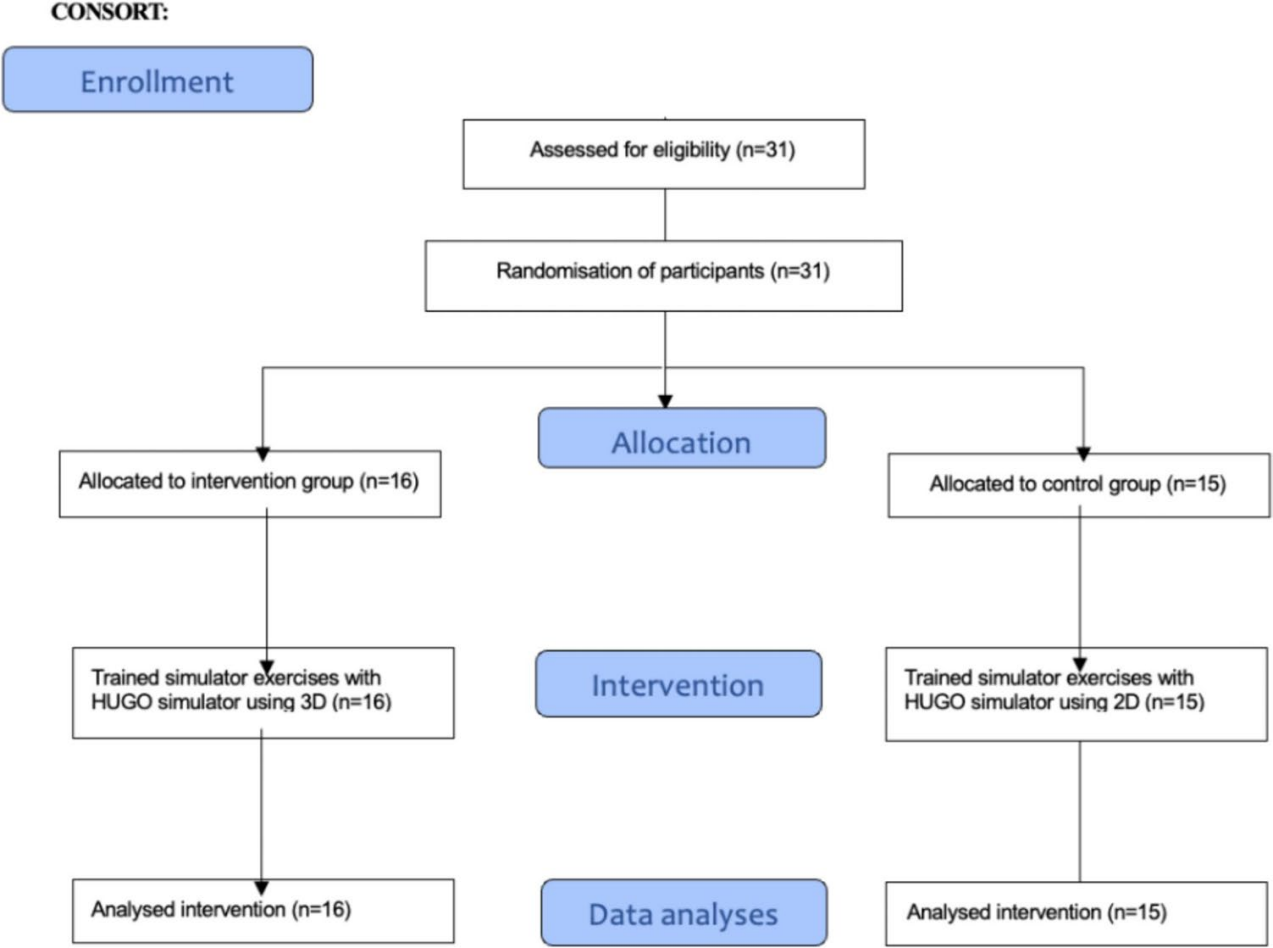
Participants flowchart flowing the CONSORT statement

We included 31 participants: 16 in the 3D and 15 in the 2D groups, with one and two stereo-blind participants in the groups. All participants completed the intervention. Participants' baseline characteristics are shown in Table 1, and the flowchart is illustrated in Fig. 2. Blinding of the participants is shown in Table 2, which shows that the blinding was only partially successful as most of the participants (22 of 31) correctly guessed which groups they were allocated to (*p* < 0.029). However, this differs from the group of stereo-blind individuals, as 2/3 guessed the allocation wrong.

**Table 1:** Participants’ baseline demographics

	3D-group (intervention) (*n* = 16)	2D-group (control) (*n* = 15)
Age, median (range)	23 (20–28)	24 (21–29)
Sex (man/woman), no	8/8	8/7
Righthanded/Lefthanded/Ambidextrous, no	13/3/0	13/1/1
Stereo blindness (Yes/no), no.^a^	15/1	13/2

^a^Defined as a score of 480 or above on TNO test Plate V + VI

**Table 2:** Results of the question on the effectiveness of intervention blinding:

	Allocate to 3D (intervention)	Allocate to 2D (control)	Total
Believed they used 3D	13	6	19
Believed they used 2D	3	9	12
Total	16	15	31

For the efficiency parameters, *Time to complete exercise and Economy of motion* (*p* < 0.001) (*p* =  < 0.001–0.003), we found that participants got better with more attempts and that the intervention group (3D) consistently outperformed the control group (2D), Table 3.

**Table 3:** Intergroup comparisons of performance over time for the efficiency parameters, *Time to complete exercise,* and* Economy of motion*

		Exercise 1—Peg board II	Exercise 2—Thread the rings	Exercise 3—Ring tower transfer	Exercise 4—Wound closure—horizontal
Time to complete exercise (s)^a^	Main effect of time	*F*(4,116) = 4.48, *p* < 0.001	*F*(4,116) = 49.90, *p* < 0.001	*F*(4,116) = 7.92, *p* < 0.001	*F*(4,116) = 136.18, *p* < 0.001
	Main effect of group	*F*(1,29) = 67.77, *p* < 0.001	*F*(1,29) = 34.64, *p* < 0.001	*F*(1,29) = 15.33, *p* < 0.001	*F*(1,29) = 17.78, *p* < 0.001
	Time x group interaction	*F*(4,116) = 0.29, *p* = 0.89	*F*(4,116) = 0.35, *p* = 0.85	*F*(4,116) = 0.55, *p* = 0.70	*F*(4,116) = 0.32, *p* = 0.87
Economy of motion (cm)^a^	Main effect of time	*F*(4,116) = 4.17, *p* = 0.003	*F*(4,116) = 10.01, *p* < 0.001	*F*(4,116) = 4.70, *p* = 0.001	*F*(4,116) = 12.71, *p* < 0.001
	Main effect of group	*F*(1,29) = 42.40, *p* < 0.001	*F*(1,29) = 13.33, *p* = 0.001	*F*(1,29) = 25.53, *p* < 0.001	*F*(1,29) = 16.06, *p* < 0.001
	Time x group interaction	*F*(4,116) = 0.90, *p* = 0.47	*F*(4,116) = 0.62, *p* = 0.65	*F*(4,116) = 0.71, *p* = 0.59	*F*(4,116) = 1.23, *p* = 0.30
Wire contact duration (s)^a^	Main effect of time	Not included for exercise	Not included for exercise	*F*(4,116) = 1.88, *p* = 0.12	Not included for exercise
	Main effect of group			*F*(1,29) = 23.27, *p* < 0.001	
	Time x group interaction			*F*(4,116) = 0.99, *p* = 0.42	

^a^Analyzed using two-way repeated measurement ANOVA on LOG-transformed data

This was consistent for all four exercises. There was no statistically significant interaction between time and intervention, meaning that the intervention group did not improve increasingly more over time compared to the control group. This was also consistent for all four exercises. For the parameter *Wire contact duration,* there was a significant effect of group (*p* < 0.001) but not of time (*p* = 0.12) or interaction of time and group (*p* = 0.42).

In contrast, for *Excessive instrument force* and *Instrument out of view*, there was only a significant improvement between the first and fifth attempts for *Excessive force* for both groups for Exercise 4. For the remaining exercises, there were no significant improvements over time (*p*-values 0.05–0.93), Table 4. For the error metrics (e.g., *Drop, Instrument collisions*), we did not find significant differences for any parameters for the four exercises except for one single parameter (*Wire Collisions*) for exercise 3 (*p* = 0.008), Table 5.

**Table 4:** Comparison of log-transformed data for 1st and 5th attempts for the parameters *Excessive instrument force and Instrument out of view*—analyzed using dependent samples *t*-test

Exercise	Parameter	Group	Attempt 1	Attempt 5	*P*-value
Exercise 1—Peg board II	Excessive instrument force (s)	3D (*n* = 16)	0.09 (0.17)	0.02 (0.03)	0.11
		2D (*n* = 15)	0.18 (0.37)	0.03 (0.05)	0.15
	Instruments out of view (s)	3D (*n* = 16)	0.06 (0.23)	0.09 (0.26)	0.7
		2D (*n* = 15)	0.3 (0.5)	0.29 (0.48)	0.93
Exercise 2—Thread the rings	Excessive instrument force (s)	3D (*n* = 16)	0.43 (0.41)	0.25 (0.29)	0.08
		2D (*n* = 15)	0.68 (0.47)	0.55 (0.42)	0.36
	Instruments out of view (s)	3D (*n* = 16)	0.25 (0.51)	0.26 (0.39)	0.87
		2D (*n* = 15)	0.82 (0.57)	0.76 (0.5)	0.58
Exercise 3—Ring tower transfer	Instruments out of view (s)	3D (*n* = 16)	0.75 (0.59)	0.63 (0.44)	0.46
		2D (*n* = 15)	1.02 (0.59)	0.73 (0.51)	0.05
Exercise 4—Wound closure—horizontal	Excessive instrument force (s)	3D (*n* = 16)	1.2 (0.57)	0.72 (0.54)	0.003*
		2D (*n* = 15)	1.54 (0.54)	1.19 (0.48)	0.02*
	Instruments out of view (s)	3D (*n* = 16)	0.76 (0.72)	0.5 (0.5)	0.12
		2D (*n* = 15)	1.1 (0.61)	0.9 (0.73)	0.22

*Statistically significant values

**Table 5:** Intergroup comparison of the cumulative error parameter score for all five attempts of the four exercises

Exercise	Parameter	Intervention (3D)	Control (2D)	*P*-value
Exercise 1—Peg board II	Drops (no.)	1.5 (1–3.5)	3 (2–4)	0.14
	Instrument collisions (no.)	14 (9.25–19)	17 (12.5–28.5)	0.12
Exercise 2—Thread the rings	Drops (no.)	4 (2–10.25)	6 (3–7)	0.57
	Instrument collisions (no.)	61 (42.25–66.5)	71 (38–80.5)	0.26
Exercise 3—Ring tower transfer	Drops (no.)	4 (2.75–7)	5 (5–10.5)	0.07
	Instrument collisions (no.)	3.5 (0–9.5)	9 (3.5–12)	0.09
	Wire collisions (no.)	96 (80–112.5)	120 (108.5–131.5)	0.008*
	Tower knockoffs (no.)	3 (1–6.25)	4 (1–8)	0.40
Exercise 4—Wound closure—horizontal	Instrument collisions (no.)	41.5 (29.25–66.5)	78 (27–122)	0.12
	Missed targets (no.)	31.5 (25.5–48.75)	59 (23–100)	0.12

All values are listed as median and interquartile range

*Statistically significant values

In contrast, the Visual Discomfort questionnaire demonstrated that the intervention (3D) group experienced significantly more difficulty focusing than the control (2D) group (*p* = 0.001) during the first session of the study but not during the second session (*p* = 0.07). None of the other side effects (dizziness, eye strain, headache, or nausea) significantly differed between the two groups in either session, Table 6. A few participants in both groups noted that they had experienced discomfort in the neck/shoulder or the wrist or thumbs.

**Table 6:** Intergroup comparison of Visual Discomfort questionnaire after each session

Training session	Question	3D	2D	*P*-values
1	Did you feel dizziness?	1 (1–1)	1 (1–1.5)	0.71
	Did your eyes feel strain?	2 (1,75–3)	1 (1–2)	0.12
	Did you feel headache?	1 (1–1)	1 (1–1)	0.57
	Did you feel nausea?	1 (1–1)	1 (1–1)	1.00
	Did you feel difficulty focusing?	2 (2–3)	1 (1–1)	0.001*
2	Did you feel dizziness?	1 (1–1)	1 (1–1)	0.95
	Did your eyes feel strain?	2 (1–3)	1 (1–2)	0.15
	Did you feel headache?	1 (1–1)	1 (1–1)	0.74
	Did you feel nausea?	1 (1–1)	1 (1–1)	0.83
	Did you feel difficulty focusing?	2 (1–3)	1 (1–1)	0.07

Values are reported using the median and interquartile range. Intergroup comparisons were done using the Mann–Whitney *U* test

*Statistically significant values

## Discussion

We found that 3D monitors improved performance compared with 2D monitors in an open console robotic system with articulated instruments. 3D monitors improve efficiency regarding speed and instrument movements as 3D has the advantages of better depth perception/spatial location, allowing for more precise movements and reducing task time [4–6]. However, we did not find any effect on errors and instrument collisions. Previous studies have also demonstrated that 3D technology significantly influences time and precision [4–6]. Badani et al. and Zwart et al. used experienced surgeons to test their hypotheses [4, 5]. This introduces a potential bias in the comparison due to the surgeons' broad spectrum of experience levels. Badani et al. required surgeons to have more than 6 months of experience. In contrast, Zwart et al. included a mix of surgeons and surgical residents with varying unknown levels of experience performing pancreatoduodenectomy anastomoses [4, 5]. Similar to these studies, our study also reveals a significant improvement in time and precision. We used only surgical novices to ensure that the intervention and control groups were comparable in experience level. Although our study only examined novices, it is conceivable that the same results might apply to experienced surgeons. It would, therefore, be interesting to investigate this outcome for experienced surgeons as well.

Another benefit of our study lies in using automated and standardized metrics. In contrast, the two aforementioned studies relied on human raters to assess surgeons' performance, which introduces a risk of bias.

Additionally, we tested the participants for stereo blindness before the randomization and stratified participants based on this. The prevalence of stereo blindness in the general population varies greatly from 1 to 30% [14]. People with stereo blindness have difficulty estimating distance, which makes it even more challenging to use 3D vision and will result in a prolonged learning curve for robot-assisted surgery [15, 16]. This allocation was performed to avoid the risk of having a majority of stereo-blind participants in any of the groups. We also attempted to blind the participants; however, the blinding was not wholly successful, as 2/3 of the participants correctly guessed which group they were allocated to. Quite a few participants who correctly guessed to be in the 3D group reported having trouble focusing at the beginning of the study, which is why they thought themselves to belong to the 3D group. Most of the other participants found it challenging to know which group they belonged to, resulting in many randomly guessing whether they were in the 3D or 2D group. Given that several studies indicate a significant advantage of operating in 3D over 2D, it would be of interest to investigate whether it would confer benefits in the collaboration between surgeons and assistants if the assistants also utilized 3D glasses in the operating room, in contrast to the current procedure where only the surgeon performs the intervention in 3D.

No difference in visual discomfort was observed between the two groups, except for the item of ‘difficulty focusing,’ where the 3D group was more challenged in the initial session. However, this difference was only observed in the first session. Similar studies comparing 3D with 2D in laparoscopy yielded outcomes consistent with ours. Additionally, they investigated visual discomfort parameters such as eye strain, dizziness, and headache, finding no disparity in the frequency of these symptoms between the two groups [17, 18]. Consequently, 3D does not induce more 'visual and physical discomfort' than 2D, supporting the adoption of 3D technology as this group operates more swiftly and precisely. A meta-analysis by Molle et al. concluded differently, as it showed that surgeons using 2D systems reported more visual discomfort compared with 3D [19]. This suggests that 3D systems do not cause more discomfort than 2D and possibly even less, which supports further implementation if 3D can reduce discomfort and improve efficiency and speed.

The sample size is a limitation of the study; however, our results indicate that the sample size was sufficient. We used medical students without robotic experience, and we cannot exclude the possibility that our results would have been different if we had used more experienced surgeons instead. We only examined five attempts on four different exercises, and it would have been interesting to see if the difference persisted with more attempts. Finally, we used a set of exercises on a virtual reality simulator to get standardized performance parameters. However, we could also have tested the effect of 3D on exercises using the real robotic system on a physical model, and we cannot exclude that there would be a different effect in this setting. We did not use a proficiency-based intervention, so we cannot say whether the effect of 3D monitors is reduced over time when participants reach proficiency.

## Conclusion

3D monitors for an open robotic console improve speed and reduce instrument movements compared with 2D monitors in a simulated setting when working with articulated instruments. We did not see any effect on error parameters overall. Furthermore, we did not identify any increased occurrence of eye strain, dizziness, headache, or other side effects, except for difficulty focusing, which only occurred initially for the 3D group.

## Supplementary Information

Below is the link to the electronic supplementary material.

Supplementary file1 (DOCX 17 KB)

Supplementary file2 (DOCX 16 KB)
