# Supplementary material for: 3D monitors improve performance on the HUGO™ RAS system: a randomised trial
Source: Surg Endosc. 2024 Oct 3;38(12):7165–71. doi: 10.1007/s00464-024-11275-y (PMC11615040; doi:10.1007/s00464-024-11275-y)
Supplement: Supplementary file 1 — Supplementary file1 (DOCX 17 KB) [file 464_2024_11275_MOESM1_ESM.docx]

Supplementary 1a – Summarized data used for Table 3. All values are listed in means and standard deviation.

| Simulator exercise | Performance parameter | Group | Attempt 1 | Attempt 2 | Attempt 3 | Attempt 4 | Attempt 5 |
| --- | --- | --- | --- | --- | --- | --- | --- |
| Peg board II | Time to Complete Exercise (seconds) | 3D | 118.42 (25.12) | 103.49 (31.83) | 106.53 (36.44) | 92.79 (30.64) | 88.7 (28.28) |
|  |  | 2D | 204.14 (58.86) | 177.56 (37.77) | 171.95 (36.49) | 161.62 (34.89) | 164.9 (53.17) |
|  | Economy of motion (centimeters) | 3D | 263.01 (53.8) | 235.74 (47.1) | 240.36 (55.57) | 232.98 (61.73) | 216.99 (36.25) |
|  |  | 2D | 394.53 (146.98) | 387.24 (113.21) | 344.87 (75.46) | 335.93 (82.93) | 349.37 (98.24) |
| Thread the rings | Time to Complete Exercise (seconds) | 3D | 202.23 (54.49) | 196.61 (57.69) | 189.75 (57.2) | 171.44 (51.68) | 156.59 (49.42) |
|  |  | 2D | 386.7 (114.96) | 321.73 (99) | 286.77 (67.61) | 296.73 (83.04) | 263.29 (69.38) |
|  | Economy of motion (centimeters) | 3D | 341.4 (155.24) | 331.86 (129.33) | 308.78 (116.69) | 284.27 (92.55) | 262.25 (76.26) |
|  |  | 2D | 544.26 (218.44) | 501.29 (200.68) | 421.16 (118.97) | 434.24 (181.84) | 398.51 (166.95) |
| Ring tower transfer | Time to Complete Exercise (seconds) | 3D | 182.64 (61) | 173.6 (69.78) | 150.34 (55.11) | 147.48 (58.48) | 143 (55.16) |
|  |  | 2D | 283.86 (85.15) | 230.32 (77.15) | 212.95 (56.29) | 228.04 (79.6) | 200.38 (64.21) |
|  | Economy of motion (centimeters) | 3D | 231.62 (79.51) | 242.99 (93.33) | 204.76 (66.58) | 204.52 (62.94) | 197.17 (47.76) |
|  |  | 2D | 340.18 (72.76) | 320.25 (137.56) | 282.25 (40.92) | 308.46 (112.27) | 254.71 (56.18) |
|  | Wire Contact Duration (seconds) | 3D | 29.74 (23.31) | 33.01 (34.47) | 22.89 (16.57) | 22.34 (22.92) | 17.59 (12.41) |
|  |  | 2D | 78.83 (50.12) | 67.71 (60.74) | 65.11 (38.03) | 78.05 (88.67) | 83.45 (77.97) |
| Wound closure - Horizontal | Time to Complete Exercise (seconds) | 3D | 285.61 (89.71) | 224.56 (66.76) | 201.18 (60.27) | 213.75 (58.07) | 165.5 (55.8) |
|  |  | 2D | 500.76 (184.09) | 406.97 (185.08) | 421.58 (189.9) | 337.72 (158.05) | 291.93 (107.49) |
|  | Economy of motion (centimeters) | 3D | 385.09 (164.28) | 319.11 (94.26) | 290.36 (104.67) | 310.84 (137.97) | 238.68 (116.13) |
|  |  | 2D | 618.89 (295.68) | 499.03 (209.57) | 517.87 (224.91) | 429.46 (188.1) | 398.35 (129.93) |
