# Supplementary material for: 3D monitors improve performance on the HUGO™ RAS system: a randomised trial
Source: Surg Endosc. 2024 Oct 3;38(12):7165–71. doi: 10.1007/s00464-024-11275-y (PMC11615040; doi:10.1007/s00464-024-11275-y)
Supplement: Supplementary file 2 — Supplementary file2 (DOCX 16 KB) [file 464_2024_11275_MOESM2_ESM.docx]

**Supplementary 1b**

| Simulator exercise | Performance parameter | Group | Attempt 1 | Attempt 2 | Attempt 3 | Attempt 4 | Attempt 5 |
| --- | --- | --- | --- | --- | --- | --- | --- |
| Peg board II | Time to Complete Exercise (LOG) | 3D | 2.07 (0.09) | 2 (0.12) | 2.01 (0.14) | 1.95 (0.13) | 1.94 (0.12) |
|  |  | 2D | 2.3 (0.12) | 2.24 (0.1) | 2.23 (0.09) | 2.2 (0.09) | 2.2 (0.13) |
|  | Economy of motion (LOG) | 3D | 2.41 (0.08) | 2.37 (0.08) | 2.37 (0.09) | 2.36 (0.1) | 2.33 (0.07) |
|  |  | 2D | 2.58 (0.13) | 2.57 (0.12) | 2.53 (0.09) | 2.52 (0.1) | 2.53 (0.11) |
| Thread the rings | Time to Complete Exercise (LOG) | 3D | 2.29 (0.13) | 2.28 (0.13) | 2.26 (0.13) | 2.22 (0.12) | 2.18 (0.11) |
|  |  | 2D | 2.57 (0.13) | 2.49 (0.14) | 2.45 (0.1) | 2.46 (0.12) | 2.41 (0.11) |
|  | Economy of motion (LOG) | 3D | 2.5 (0.17) | 2.49 (0.16) | 2.47 (0.13) | 2.43 (0.14) | 2.4 (0.12) |
|  |  | 2D | 2.71 (0.17) | 2.67 (0.17) | 2.61 (0.11) | 2.61 (0.16) | 2.57 (0.15) |
| Ring tower transfer | Time to Complete Exercise (LOG) | 3D | 2.24 (0.14) | 2.21 (0.17) | 2.16 (0.15) | 2.14 (0.16) | 2.13 (0.15) |
|  |  | 2D | 2.44 (0.13) | 2.34 (0.13) | 2.32 (0.11) | 2.33 (0.16) | 2.28 (0.16) |
|  | Economy of motion (LOG) | 3D | 2.35 (0.13) | 2.36 (0.15) | 2.3 (0.12) | 2.3 (0.12) | 2.29 (0.1) |
|  |  | 2D | 2.52 (0.09) | 2.48 (0.14) | 2.45 (0.07) | 2.47 (0.14) | 2.4 (0.1) |
|  | Wire Contact Duration (LOG) | 3D | 1.38 (0.32) | 1.34 (0.44) | 1.25 (0.41) | 1.18 (0.46) | 1.14 (0.41) |
|  |  | 2D | 1.82 (0.28) | 1.73 (0.29) | 1.75 (0.25) | 1.72 (0.37) | 1.77 (0.38) |
| Wound closure - Horizontal | Time to Complete Exercise (LOG) | 3D | 2.44 (0.15) | 2.34 (0.12) | 2.29 (0.12) | 2.32 (0.11) | 2.2 (0.12) |
|  |  | 2D | 2.68 (0.14) | 2.57 (0.19) | 2.59 (0.19) | 2.49 (0.18) | 2.44 (0.15) |
|  | Economy of motion (LOG) | 3D | 2.55 (0.18) | 2.49 (0.12) | 2.44 (0.14) | 2.46 (0.16) | 2.35 (0.16) |
|  |  | 2D | 2.75 (0.19) | 2.66 (0.18) | 2.68 (0.19) | 2.6 (0.18) | 2.58 (0.15) |
